# Supplementary material for: Aligning Large Language Models for Enhancing Psychiatric Interviews Through Symptom Delineation and Summarization: Pilot Study
Source: JMIR Form Res. 2024 Oct 24;8:e58418. doi: 10.2196/58418 (PMC11544339; doi:10.2196/58418)
Supplement: Multimedia Appendix 1 [file formative_v8i1e58418_app1.pdf]

Multimedia Appendix 1: Categories of mental disorders and the corresponding symptom labels

Category of mental disorders	Symptom labels
PTSD	re-experiencing, avoidance, negative alterations in cognition, negative alterations in emotion, exaggerated arousal and reactivity, dissociation
C-PTSD	negative self-concept, difficulty in maintaining interpersonal relationships, emotional dysregulation
Major depressive episodes	depressed mood, loss of interest, decrease in appetite, increase in appetite, insomnia, hypersomnia, psychomotor agitation, psychomotor retardation, fatigue, feelings of worthlessness, excessive guilt, difficulty in concentration, impaired memory, impaired judgment, suicidal ideation, suicide planning, and suicide attempt.
Panic attack	general anxiety, heart palpitations, sweating, trembling, shortness of breath, choking, chest pain, nausea, dizziness, chills or heat sensations, paresthesia, dissociation, loss of control, and fear of dying
Alcohol use disorder	alcohol dependence, alcohol tolerance, alcohol withdrawal

Table S1: Categories of mental disorders and the corresponding symptom labels
