# Supplementary material for: Aligning Large Language Models for Enhancing Psychiatric Interviews Through Symptom Delineation and Summarization: Pilot Study
Source: JMIR Form Res. 2024 Oct 24;8:e58418. doi: 10.2196/58418 (PMC11544339; doi:10.2196/58418)
Supplement: Multimedia Appendix 2 [file formative_v8i1e58418_app2.pdf]

# Multimedia Appendix 4: Details of the Experimental Settings

## Model

We mainly experiment with GPT-4 Turbo model (`gpt-4-1106-preview`) utilizing OpenAI's API [52] for zero-shot inference and few-shot learning. Hyperparameters [53] are set as default values: (1) `frequency_penalty` defaults to 0, (2) `logit_bias` defaults to null, (3) `logprobs` defaults to false, (4) `n` defaults to 1, (5) `presence_penalty` defaults to 0, (6) `stop` defaults to null, (7) `stream` defaults to false, (8) `temperature` defaults to 1, and (9) `top_p` defaults to 1. For fine-tuning, we also use GPT-3.5 Turbo model (`gpt-3.5-turbo-1106`). At the validation step, we perform hyperparameter selection in 12 different settings. The options are detailed as follows:

### Hyperparameter settings

- `n_epochs`: $\in \{3, 5, 10\}$
- `learning_rate_multiplier`: $\in \{0.05, 0.1, 0.2, default\}$

Based on validation results, we choose `n_epochs` as 5 and `learning_rate_multiplier` as $default$.

## Metric

### Delineating psychiatric symptoms

In this study, we employ four distinct metrics [33] to evaluate the performance of LLMs in delineating the symptoms from transcriptions. These metrics are namely: (1) `Accuracy`, (2) `Precision`, (3) `Recall`, and (4) `F1-Measure`. To evaluate the performance of LLMs in estimating the negative segments, we employ `Negative Predictive Values`. They are calculated as follows for a multi-label dataset $D$, which consists of $M = 512$ multi-label examples $(T_i, Y_i)$, and where $1 \leq i \leq M$. In this dataset, $T_i$ represents a transcription segment, and $Y_i$ denotes the corresponding set of ground-truth symptom labels (*e.g.* [ncog, reex]). The label set is denoted as $\mathcal{L}$ with $|\mathcal{L}| = 43$. We define $Z_i$ as the estimated symptom label set predicted by the LLM for the transcription segment $T_i$.

`Accuracy`: Accuracy for each segment is calculated as the ratio of correctly predicted labels to the total number of labels (both predicted and actual) for that segment. The overall accuracy is then computed as the mean of these ratios across all segments:

$$\text{Accuracy} = \frac{1}{M} \sum_{i=1}^{M} \frac{|Y_i \cap Z_i|}{|Y_i \cup Z_i|}$$

`Precision(PPV)`: Precision is defined as the ratio of correctly estimated labels to the total number of estimated symptom labels. This metric is averaged over all segments:

$$\text{Precision} = \frac{1}{M} \sum_{i=1}^{M} \frac{|Y_i \cap Z_i|}{|Z_i|}$$

`Recall`: Recall measures the ratio of correctly estimated labels to the total number of ground-truth labels, averaged across all segments:

$$\text{Recall} = \frac{1}{M} \sum_{i=1}^{M} \frac{|Y_i \cap Z_i|}{|Y_i|}$$

`F1-Measure`: F1-Measure is the harmonic mean of precision and recall, providing a balance between these two metrics. It is computed for each segment and then averaged:

$$\text{F1-Measure} = \frac{1}{M} \sum_{i=1}^{M} \frac{2\,|Y_i \cap Z_i|}{|Y_i| + |Z_i|}$$

`Negative Predictive Values(NPV)`: NPV is defined as the ratio of correctly estimated negative segments to the total number of segments estimated as negative. Note that $I(\cdot)$ denotes an indicator function. This metric is calculated across all segments:

$$\text{NPV} = \frac{\sum_{i=1}^{M} I((Y_i = \phi) \cap (Z_i = \phi))}{\sum_{i=1}^{M} I(Z_i = \phi)}$$
