# Supplementary material for: Aligning Large Language Models for Enhancing Psychiatric Interviews Through Symptom Delineation and Summarization: Pilot Study
Source: JMIR Form Res. 2024 Oct 24;8:e58418. doi: 10.2196/58418 (PMC11544339; doi:10.2196/58418)
Supplement: Multimedia Appendix 3 [file formative_v8i1e58418_app3.pdf]

Multimedia Appendix 2: Histograms of the recall mid-token distances measured for LLMs

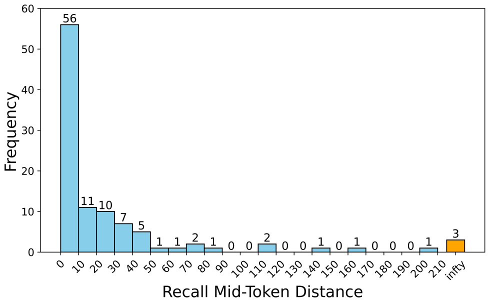

(a) In-context learning method utilizing GPT-4 Turbo model

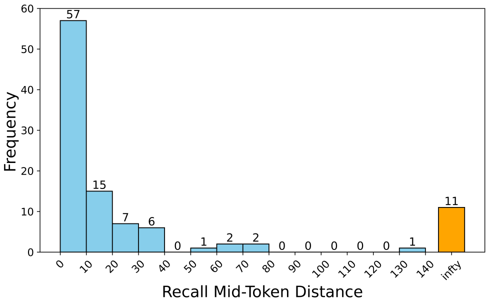

(b) Zero-shot inference utilizing GPT-4 Turbo model

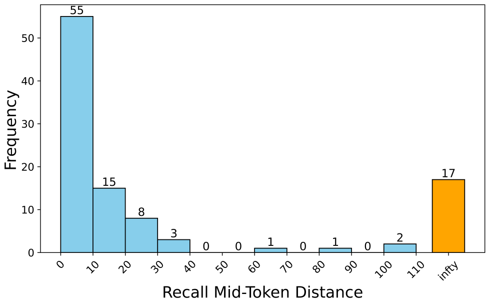

(c) Zero-shot inference with RAG utilizing GPT-4 Turbo model

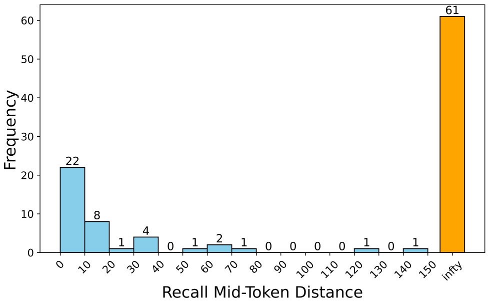

(d) Fine-Tuning method utilizing GPT-3.5 Turbo model

Figure S1: Histograms of the recall mid-token distances measured for LLMs in delineating the symptom-related sections. We evaluate the recall mid-token distance across 102 transcript segments.
