# Supplementary material for: Aligning Large Language Models for Enhancing Psychiatric Interviews Through Symptom Delineation and Summarization: Pilot Study
Source: JMIR Form Res. 2024 Oct 24;8:e58418. doi: 10.2196/58418 (PMC11544339; doi:10.2196/58418)
Supplement: Multimedia Appendix 4 [file formative_v8i1e58418_app4.docx]

|  | Task | Symptom estimation | | Section estimation | | | | |
| --- | --- | --- | --- | --- | --- | --- | --- | --- |
|  | Metric | Accuracy ($\uparrow$) | | Average of finite recall mid-token distance $d$ ($\downarrow$) | Ratio of the absence of the estimated sections, i.e., when$d=\infty$ ($\downarrow$) | Average of finite recall mid-token distance $d$ ($\downarrow$) | Ratio of the absence of the estimated sections, i.e., when$d=\infty$ (  $\downarrow$) |  |
| Model | Method | All segments | Positive segments | All segments | | Positive segments | |  |
|  |  |  |  |  |  |  |  |  |
| GPT-3.5 Turbo | Fine-tuning | 0.82 士 0.00 | 0.21 士 0.01 | — | — | 24.61 士 2.84 | 0.60 士 0.00 |  |
| GPT-4 Turbo | Zero-shot | 0.64 士 0.00 | 0.33 士 0.03 | — | — | 13.14 士 0.53 | 0.11 士 0.02 |  |
| GPT-4 Turbo | In-context learning | 0.54 士 0.01 | 0.27 士 0.01 | — | — | 22.88 士 1.49 | 0.03 士 0.00 |  |
