# Supplementary material for: Aligning Large Language Models for Enhancing Psychiatric Interviews Through Symptom Delineation and Summarization: Pilot Study
Source: JMIR Form Res. 2024 Oct 24;8:e58418. doi: 10.2196/58418 (PMC11544339; doi:10.2196/58418)
Supplement: Multimedia Appendix 5 [file formative_v8i1e58418_app5.pdf]

Multimedia Appendix 5: Prompts Used in Experiments

Prompt for zero-shot inference

> - messages for system: "You will be given an interview. When answering the psychiatric symptoms associated with PTSD and the section that represents them, please be sure to answer in the form [{'symptom': '...', 'section': '...'}, {'symptom': '...', 'section': '...'}, ...]. If you think there are multiple symptoms in a particular section, you can answer in the form [{'symptom': '..., ...', 'section': '...'}, ...]. If there are no psychiatric symptoms associated with PTSD in a given interview, please answer [{'symptom': 'none', 'section': 'none'}]. We'll give you a label (symptom) for the psychiatric symptoms associated with PTSD. We have the following symptoms: reex(Reexperience), avoid(Avoidance), ncog(Negative change in cognition), nmood(Negative change in mood), arousal(Arousal), disso(Dissociation), demo(Difficulty in emotional regulation), nself(Negative self-image), drelat(Difficulty in relationship), depress(Depressed mood), dinter(Decreased interest), dapp(Decreased appetite), iapp(Increased appetite), insom(Insomnia), hsom(Hypersomnia), agit(Psychomotor agitation), retard(Psychomotor retardation), fati(Fatigue), worth(Worthlessness), guilty(Excessive guilt), dcon(Decreased concentration), dmemo(Decreased memory), ddeci(Decreased decision), suii(Suicidal ideation), suip(Suicide plan), suia(Suicide attempt), anxiety(Anxiety), palpi(Palpitation), sweat(Sweating), trembl(Trembling), breath(Shortness of breath), chok(Choking), chest(Chest pain), nausea(Nausea), dizzy(Dizziness), chhe(Chilling), pares(Paresthesia), control(Loss of control), dying(Fear of dying), adepen(Alcohol dependence), atoler(Alcohol tolerance), awithdr(Alcohol withdrawal), and a total of 43 symptoms. When answering a symptom, be sure to use a label, and when answering a section, be sure to use the exact words from the interview."
> - messages for user:
>   - **Instruction**: "Look at the following interview and if you think that there are psychiatric symptoms associated with PTSD in the interview, please tell me the symptom and the section that represents it"
>   - **Input Query**: {A segment where we want to delineate psychiatric symptoms}

Prompt for zero-shot inference with RAG

> Answer the question based on the content below:
> {Trauma and Stressor-Related Disorders chapter of the DSM-5 book}
>
> Question: You will be given the following psychiatric symptoms associated with PTSD in the form of a label(symptom). reex(Reexperience), avoid(Avoidance), ncog(Negative change in cognition), nmood(Negative change in mood), arousal(Arousal), disso(Dissociation), demo(Difficulty in emotional regulation), nself(Negative self-image), drelat(Difficulty in relationship), depress(Depressed mood), dinter(Decreased interest), dapp(Decreased appetite), iapp(Increased appetite), insom(Insomnia), hsom(Hypersomnia), agit(Psychomotor agitation), retard(Psychomotor retardation), fati(Fatigue), worth(Worthlessness), guilty(Excessive guilt), dcon(Decreased concentration), dmemo(Decreased memory), ddeci(Decreased decision), suii(Suicidal ideation), suip(Suicide plan), suia(Suicide attempt), anxiety(Anxiety), palpi(Palpitation), sweat(Sweating), trembl(Trembling), breath(Shortness of breath), chok(Choking), chest(Chest pain), nausea(Nausea), dizzy(Dizziness), chhe(Chilling), pares(Paresthesia), control(Loss of control), dying(Fear of dying), adepen (Alcohol dependence), atoler (Alcohol tolerance), and awithdr (Alcohol withdrawal), for a total of 43 symptoms. Read the following interview transcript and extract the psychiatric symptom associated with PTSD and the section that represents it. When extracting a symptom from the interview, be sure to answer using only label except (symptom) in the form label(symptom), and when extracting a section from the interview, be sure to answer using only the given interview content. Also, when extracting a section from an interview multiple times, be sure to answer in the form of "...", "...", "...", "...". If there are no psychiatric

symptoms associated with PTSD in a given interview, answer “none”.
- Interview content: {A segment where we want to delineate psychiatric symptoms}

Answer:
- Symptom :
- Section :

Prompt for few-shot learning

- messages for system: “You will be given several sets of inputs and outputs, where the inputs are the interview transcript segments and the outputs are the psychiatric symptoms of associated with PTSD from the previous input and the sections where the symptoms appear. At the end, you will be given a transcript of the interview in Input and asked to identify the psychiatric symptoms associated with PTSD and the section in which the symptom appears, using the form [{‘symptom’: ‘...’, ‘section’: ‘...’}, {‘symptom’: ‘...’, ‘section’: ‘...’}, ...]. If you think there are multiple symptoms in a particular section, you can answer in the form [{‘symptom’: ‘..., ...’, ‘section’: ‘...’}, ...]. If there are no psychiatric symptoms associated with PTSD in a given interview, answer [{‘symptom’: ‘none’, ‘section’: ‘none’}]. You can use in-context learning to answer using the previous input and output sets. I’ll give you a label(symptom) for a psychiatric symptom associated with PTSD. We have the following symptoms. reex(Reexperience), avoid(Avoidance), ncog(Negative change in cognition), nmood(Negative change in mood), arousal(Arousal), disso(Dissociation), demo(Difficulty in emotional regulation), nself(Negative self-image), drelat(Difficulty in relationship), depress(Depressed mood), dinter(Decreased interest), dapp(Decreased appetite), iapp(Increased appetite), insom(Insomnia), hsom(Hypersomnia), agit(Psychomotor agitation), retard(Psychomotor retardation), fati(Fatigue), worth(Worthlessness), guilty(Excessive guilt), dcon(Decreased concentration), dmemo(Decreased memory), ddeci(Decreased decision), suii(Suicidal ideation), suip(Suicide plan), suia(Suicide attempt), anxiety(Anxiety), palpi(Palpitation), sweat(Sweating), trembl(Trembling), breath(Shortness of breath), chok(Choking), chest(Chest pain), nausea(Nausea), dizzy(Dizziness), chhe(Chilling), pares(Paresthesia), control(Loss of control), dying(Fear of dying), adepen(Alcohol dependence), atoler(Alcohol tolerance), awithdr(Alcohol withdrawal), and a total of 43 symptoms. When answering a symptom, be sure to answer with a label, and when answering a section, be sure to answer with the exact wording of the interview.”
- messages for user:
  - **Instruction**: “Based on the correspondence between the given input and output examples, if you think the interview in the last input has a psychiatric symptom associated with PTSD, provide the symptom and the section that represents it.”
  - **In-context example**:
    * Transcript segment: “I: Your head hurts. P4: I have some headaches, I have some dizziness or something like that, I just have a bad headache, and then I don’t know why I can’t eat or anything, just.”
    * Ground-truth label: [{‘symptom’: ‘dizzy’, ‘section’: ‘I have some dizziness or something’}]
  - **Input Query**: {A segment where we want to delineate psychiatric symptoms}

Prompt for fine-tuning

[Prompt for fine-tuning]

- messages for system: “You will be given several sets of inputs and outputs, where the inputs are the interview transcript segments and the outputs are the psychiatric symptoms of associated with PTSD from the previous input and the sections where the symptoms appear. If you think there are multiple symptoms in a particular section, you can answer in the form [{‘symptom’: ‘...,

...', 'section': '...'}, ...]. If there are no psychiatric symptoms associated with PTSD in a given interview, answer [{'symptom': 'none', 'section': 'none'}]. When answering a symptom, be sure to answer with a label, and when answering a section, be sure to answer with the exact wording of the interview."

- messages for user:
  - **Instruction**: "Look at the following interview and if you think that there are psychiatric symptoms associated with PTSD in the interview, please tell me the symptom and the section that represents it."
  - **Input Query**: "I: Your head hurts. P4: I have some headaches, I have some dizziness or something like that, I just have a bad headache, and then I don't know why I can't eat or anything, just."
- messages for assistant :
  - **Ground-truth label**: [{'symptom': 'dizzy', 'section': 'I have some dizziness or something'}]

[Prompt for inference on fined-tuned model]

- messages for system: "You will be given several sets of inputs and outputs, where the inputs are the interview transcript segments and the outputs are the psychiatric symptoms of associated with PTSD from the previous input and the sections where the symptoms appear. If you think there are multiple symptoms in a particular section, you can answer in the form [{'symptom': '..., ...', 'section': '...'}, ...]. If there are no psychiatric symptoms associated with PTSD in a given interview, answer [{'symptom': 'none', 'section': 'none'}]. When answering a symptom, be sure to answer with a label, and when answering a section, be sure to answer with the exact wording of the interview."
- messages for user:
  - **Instruction**: "Look at the following interview and if you think that there are psychiatric symptoms associated with PTSD in the interview, please tell me the symptom and the section that represents it."
  - **Input Query**: {A segment where we want to delineate psychiatric symptoms}
