# Supplementary material for: Aligning Large Language Models for Enhancing Psychiatric Interviews Through Symptom Delineation and Summarization: Pilot Study
Source: JMIR Form Res. 2024 Oct 24;8:e58418. doi: 10.2196/58418 (PMC11544339; doi:10.2196/58418)
Supplement: Multimedia Appendix 6 [file formative_v8i1e58418_app6.pdf]

Multimedia Appendix 3: Comparison of the summaries generated by human experts, GPT-4 Turbo model and GPT-4 Turbo model using RAG.

Human Expert	
Reflects on growing up in North Korea as a single mother, experiencing great economic hardship and adversity, including her brother being captured while traveling to and from China. In 1977, her brother's imprisonment and a visit from Kim Il-sung led to his exile from the city to the countryside. Despite his academic achievements, he was unable to continue his education after dropping out of high school and was forced to work on a farm. As a result of this, she felt that she had no hope and prospects for the future, did not want to live, and was miserable, and resented her brother and mother a lot, and although her peers talked about her brother's problems behind her back, she did not experience bullying. In 1992, he moved to a tobacco farm and began farming, which improved his living conditions somewhat, but he was always worried that he would be deported back to a poor environment. During the hardship march, he said he felt physically exhausted, uncertain about the future, and threatened for his life because there was no food, and he often saw his neighbors starve to death. Due to economic problems, the entire family fled to China in 1999, and for the next five years they stayed in China, experiencing constant anxiety and heart palpitations related to North Korea. On four occasions, she was abruptly separated from her children when they were unable to go into hiding, was sent back to China to be held in guard houses and training centers, and witnessed verbal and physical violence against others. This resulted in physical weakness, increased interpersonal wariness, and feelings of helplessness, regret, and confusion, but she did not have suicidal thoughts because of her children. Came to South Korea around 2004 and worked as a sewing machine operator and caregiver. Experienced generalized happiness while living with her family and was able to express herself freely, unlike in North Korea. She has not experienced any trauma symptoms that affect her daily life, but she has experienced re-experiencing symptoms such as dreams of being repatriated, being arrested and chased by the Bureau, dreams of lying down with someone dead, and breaking out in cold sweats. The frequency of her nightmares was high in the early days of her defection, but has gradually decreased, and she is no longer surprised when she has nightmares. However, she finds it difficult to get the memories of North Korea out of her head, especially those related to her brother, and tries to avoid mentioning them because she feels emotionally distressed.	
**GPT-4**	**GPT-4 with RAG**
P9 experienced extreme mental and physical stress in North Korea and China. She faced natural disasters, extreme hunger, arrest of her family, and constant threats from public security, which led to fears of deportation and loss of her family. Experiential trauma manifested as regular nightmares, restless sleep, anxiety, avoidance, arousal, re-experiencing symptoms, and extreme stress reactions. She experienced severe hunger during the "hardship march" in North Korea, and legal insecurity and fear of family separation in China. These events led to her escape to South Korea and continue to impact her life today. The trauma recurs during the day and at night, and she continues to be extremely worried about her and her family's safety. Overt symptoms of mental health include anxiety, depression, suicidal thoughts, death-related dreams, overreaction in everyday life, and physical symptoms such as increased heart rate and sweating. These symptoms meet the criteria for PTSD, and Patient P9 is highly traumatized and in need of professional mental health care.	The interviewee experienced extreme trauma as a result of the difficult life in North Korea, the defection process, arrest in China, and threats of repatriation. Direct threats to their lives and the lives of their families, abuse, and forced relocation contributed to their trauma and mental distress. Natural disasters, painful experiences of abuse, and separation from their families caused ongoing psychological stress, which led to nightmares and re-experiencing of trauma even after arriving in South Korea. These experiences have left deep emotional sequelae, and the interviewee complains of severe emotional difficulties, such as, for example, constant anxiety, pining feelings, alienation, fear of death, and loss of self-confidence. However, there is no mention of specific symptoms associated with PTSD in the interview, suggesting that further evaluation by a psychiatrist is needed for a diagnosis.

Table S1: Comparison of the summaries generated by human experts, GPT-4 Turbo model and GPT-4 Turbo model using RAG. The summary is based on the transcript of the interview with patient P9.
