# Supplementary material for: Aligning Large Language Models for Enhancing Psychiatric Interviews Through Symptom Delineation and Summarization: Pilot Study
Source: JMIR Form Res. 2024 Oct 24;8:e58418. doi: 10.2196/58418 (PMC11544339; doi:10.2196/58418)
Supplement: Multimedia Appendix 7 [file formative_v8i1e58418_app7.pdf]

Multimedia Appendix 6: Additional Experiments on Simulated Data generated by LLMs

Generation of Simulated Data

We generate simulated interview data between a therapist (denoted by I) and a North Korean defector with PTSD (denoted by P77) based on the paper titled "Cactus: Towards Psychological Counseling Conversations using Cognitive Behavioral Theory" [1], which aims to provide realistic multi-turn conversations by having the AI counselor. We follow the method presented in the paper and the pipeline provided in the github repository to generate the simulated data. Additionally, the temperature parameter is set to 0.7 for this part. For the North Korean defector with PTSD, the stressor and symptom summary label for P14, used in our summarization task, is also used in the intake form as "present problem". We use this generated simulated interview data as test data, while the training and validation datasets and other experimental settings remain consistent with the original experiment setup. The simulated data consists of 18 transcript segments, and after labeling by mental health professionals, 7 of these segments contain ground-truth symptoms and section labels. The simulated data is as follows:

> I: Hi Tristan, I'm glad you're here today. How are you feeling about starting this counseling session?
> P77: I'm feeling a bit nervous but also hopeful. I've been dealing with a lot, and I guess I'm ready to talk about it.
> I: It's completely normal to feel a mix of nerves and hope when starting this journey. Since you mentioned feeling overwhelmed, I think a good place for us to start might be to explore these feelings of worthlessness you've described. Could you tell me more about when you tend to experience these feelings most strongly?
> P77: Well, the feelings of worthlessness come up a lot, especially when I think about my family back in North Korea and their suffering because of decisions I've made. It's like I can't shake the guilt, and it makes me feel I'm failing as a family member, even though I'm here trying to provide for them. Sometimes, it just seems like whatever I do isn't enough, which really weighs on me.
> I: Thank you for sharing that with me, Tristan. It sounds incredibly challenging to carry that sense of guilt and responsibility for your family's situation. It's clear you care deeply about them. When these feelings of not doing enough come up, what tends to go through your mind? Let's explore these thoughts and see if we can address the intensity of these feelings.
> P77: It's like my mind goes into a spiral, really. I start thinking that I should have done more, could have been smarter about the decisions I made. Then, I start doubting all my choices, wondering if I've ever made any right decisions at all. It feels like there's a constant voice telling me I could've avoided all the suffering if I had just been better somehow. It's exhausting, and sometimes I even question the point of trying if I'm just going to keep failing them.
> I: Thank you for being so open about what goes through your mind, Tristan. It sounds like these thoughts really take a toll on you, leading to a cycle of doubt and exhaustion. This is where the technique of decatastrophizing might be really helpful. It aims to break down these overwhelming thoughts and assess them more realistically. To start, could we look at one specific thought that often comes up for you? For example, you mentioned the thought that you should have done more. Let's analyze that thought together and see if we can challenge its validity and explore its impact on you. How does that sound?
> P77: That sounds like a plan, but honestly, it's daunting to even start dissecting these thoughts. They feel so deeply ingrained, almost like they're a part of who I am now. I'm skeptical about how much we can change that, but I'm willing to try because I'm tired of feeling this way all the time. Let's take that thought of 'I should have done more' and see where we can go with it.
> I: That's a great starting point, Tristan, and I appreciate your willingness to dive into these challenging thoughts despite the skepticism. It's completely understandable to feel daunted by the prospect of dissecting thoughts that have felt like a part of you for so long. Let's begin by examining the thought, I should have done more. Can you describe a specific situation recently where this thought came up?

[1] https://arxiv.org/pdf/2407.03103

By focusing on a particular instance, we might be able to understand the context and the emotions tied to it more clearly. This can help us to start breaking down the thought and assessing its accuracy and helpfulness.
P77: The most recent time that thought hit me was last week when I was sending money back home. I had planned to send more, but with my own expenses piling up, I couldn't manage it. That's when I started thinking, "I should have done more." I felt like I was letting them down again. It's as if no matter how much I send, it's never going to be enough to make up for not being there with them. It leaves me feeling stuck and helpless, like I'm failing them over and over.
I: Thank you for sharing that specific incident, Tristan. It definitely helps to understand the situations where these thoughts become more intense. It's clear that you're putting a lot of pressure on yourself to meet what seems like an incredibly high standard. Let's start by questioning this thought a bit: When you say I should have done more, what would have been enough in that situation? Is there a clear standard you're aiming for, or does it feel like the goalpost is always moving? Let's explore if this thought is based on a realistic expectation or if it might be an example of setting an impossible standard for yourself.
P77: It feels like the goalpost is always moving. No matter what I do, it never seems to be enough. I can't even define what 'enough' would look like. It's always just this vague notion that I should be doing more, that I should be better. I guess I've internalized that no amount of effort will truly change their situation back home, which makes me question the point of my efforts. It's like I'm trapped in this cycle of perpetual inadequacy.
I: You've articulated that feeling of a 'moving goalpost' really well, Tristan. It sounds like part of the struggle is that the criteria for 'enough' isn't just unclear—it feels unattainable. This can indeed create a cycle of perpetual inadequacy, as you've described. Let's think about this together: if the goalpost always seems to move, might it be possible that the standards you're holding yourself to are not just unrealistic, but perhaps also unkind? How might it change your perspective if you allowed yourself some flexibility in what you expect from yourself?
P77: It's hard to think about giving myself that flexibility. I mean, if I don't push myself, who will? There's always this fear that if I let up even a little, everything will just fall apart. It's like I'm the only glue holding everything together for my family, and without that, what are they left with? I guess I'm scared that by lowering my standards, I might just be giving up on them.
I: It's understandable that you feel like you need to maintain this high level of effort because you see it as a way of supporting your family. This sense of responsibility is clearly strong, and it speaks volumes about your commitment and love for them. However, it's also important to consider the sustainability of this pressure you place on yourself. Let's explore the idea that being the 'glue' doesn't necessarily mean you can't set realistic and kinder standards for yourself. What do you think might happen if you were to adjust these standards slightly? How might it affect your well-being and, in turn, your ability to care for your family?
P77: It's a bit terrifying to think about adjusting those standards because they've been my driving force for so long. But honestly, hearing you frame it as being kinder to myself rather than just lowering my standards does shift my perspective a bit. Maybe if I'm not so exhausted all the time, I could actually be more effective in helping my family, not less. It's just hard to shake off the guilt that comes with even thinking about it.
I: It's very insightful of you to recognize that alleviating some of that pressure could potentially make you more effective rather than less. Guilt is a powerful emotion, and it's not uncommon for it to arise when considering changes to deeply ingrained habits or beliefs, especially those tied to family responsibilities. Let's explore that guilt a bit further. What are the thoughts or beliefs that fuel this guilt when you think about giving yourself more leeway or kindness? Understanding these can be a key step in managing them and possibly redefining what your support for your family looks like.
P77: The guilt mostly comes from feeling like I'm abandoning them if I ease up on myself. It's like I'm betraying my own values, the ones that have been guiding me all these years to keep pushing, no matter what. Part of me knows it's not rational because being kinder to myself doesn't mean I care any less about my family. But there's another part that's just terrified of what letting go even a little could mean. It's confusing and it feels like I'm stuck between being harsh on myself and fearing the

consequences of being kinder.
I: That's a very profound reflection, Tristan. It sounds like you are caught in a challenging space between your values and the practical, emotional toll they take on you. It's important to honor these feelings and the values you hold dear, yet it's equally important to assess their impact on your well-being. Balancing these can be tough, but it's not impossible. Let's consider what small steps could look like in terms of being kinder to yourself. What might be one small change you could experiment with that feels manageable, yet could bring some relief? This doesn't have to mean a big shift but could be a gentle easing of the pressure you place on yourself.
P77: That idea of just making a small, manageable change sounds less daunting. Maybe I could start by setting aside a little time each day just for myself, to do something I enjoy, without feeling guilty about it. It might help me recharge and break away from the constant stress for a bit. It's still a bit scary to think about, but maybe it's a step toward not feeling so overwhelmed all the time.
I: That sounds like a wonderful and very achievable step, Tristan. Setting aside a little time each day for yourself can be incredibly beneficial. It not only allows you to recharge but also reinforces the idea that taking care of yourself is not a luxury—it's a necessity, and it doesn't detract from your care for your family. How do you think you might begin to implement this new routine? What are some activities that you enjoy that you could incorporate into this time for yourself?
P77: I've always enjoyed reading, and I think it would be a good start to spend some time each evening with a book. It's something I used to love before all the stress piled up. This feels like a small step, but even deciding on this feels like a bit of a relief, like I'm allowing myself a moment away from all the responsibilities. I'm curious, though, how do I deal with the guilt that might come creeping in during these moments? It's like I'm programmed to think that every minute should be productive or for others.
I: It's great to hear that you're considering incorporating reading into your daily routine, Tristan. It's a positive step towards reclaiming some of your own time and space. Regarding the guilt you mentioned, it's quite common to feel that way, especially when you've been in a long-standing pattern of prioritizing others over yourself. One strategy we can try is to reframe these moments of self-care as not just beneficial for you, but indirectly beneficial for your family as well. When you are less stressed and more replenished, you're likely to be more present and effective in your interactions with them. Another approach could be to gently remind yourself during these times that it's okay to take care of yourself, and that doing so is not a sign of neglect but of strength. It might help to visualize your self-care as an essential part of maintaining the 'glue' that holds everything together, by keeping you healthy and resilient. How does that perspective resonate with you? Would you be willing to try these reframing techniques when you start to feel guilty?
P77: That reframing technique makes sense, and I appreciate the perspective that taking care of myself is also taking care of my family. I can try to remind myself of that, though I suspect it'll be a challenge to change these ingrained thoughts. I'm willing to give it a shot, but I'm still a bit anxious about actually implementing it. It feels like stepping into unfamiliar territory, wondering if I'm just being selfish or actually doing something beneficial.
I: You're taking a courageous step by even considering these changes, Tristan. It's natural to feel anxious about stepping into unfamiliar territory, especially after a long period of putting others first. Remember, feeling selfish when you start prioritizing your own needs is common, especially for someone as devoted to their family as you are. But self-care isn't selfish; it's a critical part of being able to sustain the support you offer to others. Maybe we could think about this as an experiment at first. You're testing out a new way of being, one that includes more balance. This could allow you to observe and evaluate how these changes affect not just your own well-being but also how you engage with your family. How would you feel about approaching this as a sort of trial, to see the positive differences it might bring?
P77: Approaching this as a trial does make it seem less permanent and scary, which helps a little. I think framing it that way allows me to see it not just as a change, but as an exploration of what could actually improve my life and by extension, the quality of life for my family too. I'm still nervous about the outcome, but I'm more open to seeing where this could lead. Thank you for suggesting a perspective that makes this feel like a step forward rather than a departure from my duties.
I: That's a very constructive way to look at it, Tristan. Approaching these changes as an exploration

rather than a drastic shift can make the process feel more manageable and less overwhelming. As you start this trial, I'd encourage you to keep a journal of your thoughts and feelings. This can be a valuable tool to observe the changes you experience and the impact it has on your well-being and your interactions with your family. It might also help in addressing the anxiety about the new routine by providing a space to reflect and see the progress you're making. How do you feel about incorporating journaling into this experiment?

P77: Incorporating journaling sounds like a good idea. It might help me to see the patterns in my thoughts and feelings more clearly, and to track any changes that come from this new approach. It's still a bit intimidating to think about monitoring my inner experiences so closely, but I can see the potential benefits. I'll try to keep an open mind and see it as part of the exploration, like you mentioned.

I: That's a great attitude, Tristan. Journaling can indeed provide insightful reflections and help you to recognize the shifts in your thoughts and feelings as you embark on this journey. Starting with just a few minutes a day, whenever you feel comfortable, can make it less daunting. Writing doesn't have to be structured or perfect—it's just a personal space for your thoughts. How do you think you will start with this? Do you have any specific time of day in mind that might work best for you?

P77: I think starting my journaling in the evening would work best for me, as it would be a way to wind down and process my thoughts from the day. It's still a bit overwhelming to think about diving deep into my feelings, but I know it's necessary. I'm hoping that this will help me manage my guilt and anxiety better by making them more tangible and less daunting as I write them down.

I: That sounds like a practical plan, Tristan. Setting aside time in the evening to reflect and process your day can be a calming routine and a powerful tool for managing feelings of guilt and anxiety. As you begin this practice, try to approach it with curiosity and compassion towards yourself. Remember, it's not about judging your feelings but understanding and accepting them. How do you feel about starting this new routine tonight? What book do you think you might start reading during your personal time?

P77: I think starting tonight sounds good. I've got a book on my shelf that I've been meaning to get to for a while now—it's a novel about resilience and overcoming adversity, which feels quite fitting. I'm a bit apprehensive about initiating all these changes, but I feel somewhat relieved to have a plan in place. It's comforting to think that this might lead to a more balanced life, even if it's just a small step at a time. Thank you for helping me to see these possibilities.

I: You're very welcome, Tristan. It sounds like you've chosen a perfect book to start with, and it aligns beautifully with your journey. Reading about resilience and overcoming adversity can offer you valuable insights and inspiration. And remember, each small step you take is a significant part of your broader path to a more balanced and fulfilling life. As you embark on this journey of journaling and setting aside time for yourself, I'll be here to support you and discuss any insights or challenges you encounter. When we meet next, feel free to share how these initial steps went for you. How does that sound for our next session?

P77: That sounds very encouraging, thank you. I'm genuinely intrigued to see how these small changes might affect my overall well-being and my dynamics at home. I'll make sure to note down my experiences and feelings to share with you in our next session. It feels a bit like venturing into unknown waters, but I'm hopeful that this could lead to a better understanding of myself and perhaps a healthier way of managing my responsibilities and emotions.

I: That's the spirit, Tristan. Venturing into unknown waters can indeed be daunting, but it also holds the possibility of discovery and growth. I'm glad to hear you're open to observing and sharing how these changes play out. As you experiment with these new practices, remember that this is a process, and it's okay to adjust as needed. Your resilience and willingness to explore new approaches are strengths that will serve you well. I look forward to hearing about your experiences and reflections in our next session. If you ever feel overwhelmed or need to discuss anything before then, feel free to reach out. Take care, and I wish you a peaceful start with your new routine tonight.

P77: Thank you for your support and understanding. I feel a bit more equipped to handle this process now. It's still daunting, but knowing I can discuss my progress and setbacks with you makes it seem more manageable. I'll start with the journaling and reading tonight and see how it goes. Looking forward to sharing my experiences with you next time.

I: You're welcome, Tristan. I'm glad you're feeling a bit more prepared to start this journey. Remember,

it's perfectly normal to have mixed feelings about new changes, especially when they involve deeply ingrained habits and emotions. Take it one step at a time, and know that each step is a part of your progress, no matter how small it may seem. I'm here to help you navigate this process, and I'm looking forward to our next session where we can explore your experiences and continue working together towards a more balanced life. Have a great evening with your book, and take care until we meet again.
P77: Thank you, truly. It's comforting to know there's space to talk about these things without immediate judgment. I'll take the steps we discussed tonight and try to observe the changes, both in how I feel and how I manage my day. I'm still nervous about the adjustments, but also curious to see what comes of them. I look forward to our next session to discuss how these small changes are impacting my life.

Experimental results

Range	Frequency
$0 \leq d < 10$	6
$10 \leq d < 20$	1
$d \geq 20$	0

Table S1: Performance of zero-shot inference setting using GPT-4 Turbo model on delineating the symptom-related sections from simulated data of a North Korean Defector with PTSD, measured by the recall mid-token distance d.

Model	Method	`Accuracy`	`Precision(PPV)`	`NPV`	`Recall`	`F1-Measure`
GPT-3.5 Turbo	Fine-Tuning	0.667 ± 0.023	0.666 ± 0.023	0.917 ± 0.000	0.722 ± 0.005	0.685 ± 0.003
GPT-4 Turbo	ICL	0.485 ± 0.003	0.551 ± 0.017	1.000 ± 0.000	0.556 ± 0.013	0.541 ± 0.007
GPT-4 Turbo	Zero-Shot	0.531 ± 0.006	0.619 ± 0.012	1.000 ± 0.000	0.620 ± 0.000	0.599 ± 0.003
GPT-4 Turbo	Zero-Shot (w/ RAG)	0.614 ± 0.019	0.679 ± 0.016	1.000 ± 0.000	0.667 ± 0.013	0.661 ± 0.015

Table S2: Performance of LLMs in estimating symptoms from simulated data of a North Korean Defector with PTSD. For each setting, we report the average score and its standard deviation for 3 trials. Since we use non-zero temperature parameter of LLM, the performance varies among different trials.

	G-Eval					BERT
	Coherence	Consistency	Fluency	Relevance	Overall	Score
`Strs+Symp`	4.19	4.10	1.53	4.37	3.54	0.57
`Strs+Symp` (w/ RAG)	4.22	4.03	1.06	4.18	3.37	0.55

Table S3: Evaluation of the summaries generated by GPT-4 Turbo model on the simulated data of a North Korean Defector with PTSD : (1) G-Eval measures the coherence/consistency/fluency/relevance score of GPT's summary, and (2) BERTScore measures the similarity between the summaries obtained by GPT-4 Turbo model and a human expert. Note that the maximum score of coherence, consistency, fluency and relevance measured by G-Eval is 5,5,3, and 5, respectively, while the BERTScore ranges from 0 to 1. We evaluate summaries generated from `Strs+Symp` uses both the estimated stressors and symptoms.
